# Redox regulation of proteostasis

**DOI:** 10.1016/j.jbc.2024.107977

**Published:** 2024-11-08

**Authors:** Long Duy Duong, James D. West, Kevin A. Morano

**Affiliations:** 1 Department of Microbiology & Molecular Genetics, McGovern Medical School, The University of Texas Health Science Center at Houston, Houston, Texas, USA; 2 Biochemistry & Molecular Biology Program, Departments of Biology and Chemistry, The College of Wooster, Wooster, Ohio, USA

**Keywords:** proteostasis, redox regulation, oxidation, oxidative stress, transcriptional response, post-translational modification, thiol modification, chaperone, heat shock protein, holdase, foldase, translation repression, protein degradation

## Abstract

Oxidants produced through endogenous metabolism or encountered in the environment react directly with reactive sites in biological macromolecules. Many proteins, in particular, are susceptible to oxidative damage, which can lead to their altered structure and function. Such structural and functional changes trigger a cascade of events that influence key components of the proteostasis network. Here, we highlight recent advances in our understanding of how cells respond to the challenges of protein folding and metabolic alterations that occur during oxidative stress. Immediately after an oxidative insult, cells selectively block the translation of most new proteins and shift molecular chaperones from folding to a holding role to prevent wholesale protein aggregation. At the same time, adaptive responses in gene expression are induced, allowing for increased expression of antioxidant enzymes, enzymes that carry out the reduction of oxidized proteins, and molecular chaperones, all of which serve to mitigate oxidative damage and rebalance proteostasis. Likewise, concomitant activation of protein clearance mechanisms, namely proteasomal degradation and particular autophagic pathways, promotes the degradation of irreparably damaged proteins. As oxidative stress is associated with inflammation, aging, and numerous age-related disorders, the molecular events described herein are therefore major determinants of health and disease.

## Origins of oxidants and other reactive species that modify proteins

Accumulation of oxidants is closely correlated with aging, age-related diseases (including cancers, neurodegenerative diseases, cardiovascular diseases, and chronic inflammation), and the immune response against microbes (1, 2, 3). Many of these oxidants comprise a family of O_2_-derived reactive oxygen species (ROS). ROS can originate from multiple sources, including aberrant electron transport during oxidative phosphorylation (which leads to superoxide (•O_2_^-^) production), activation of NADPH oxidases during the oxidative burst phase of the innate immune response to produce •O_2_^-^, the action of various oxidases that contribute to oxidative protein folding and xenobiotic metabolism in the endoplasmic reticulum (that produce either hydrogen peroxide (H_2_O_2_) or •O_2_^-^), and the action of other oxidases that reside in peroxisomes (4, 5, 6). Moreover, adverse environmental conditions such as exposure to ionizing radiation or various toxicants also increase intracellular oxidant levels (7).

Certain ROS can be used in the downstream production of additional oxidants and reactive species. For instance, the chlorinating agent and oxidant hypochlorous acid (HOCl) is produced from H_2_O_2_ and chloride by myeloperoxidase, an enzyme expressed in neutrophils to combat microbial infections (8). Separately, nitric oxide (•NO), which is generated by nitric oxide synthases, can react with superoxide to generate peroxynitrite (ONOO^–^), a potent oxidant and nitrating agent (9); alternatively, •NO can undergo further reactions to generate several distinct nitrosating agents (10). Powerful oxidants like hydroxyl radical (•OH) and ONOO^–^ can also react with polyunsaturated fatty acids in glycerophospholipids to generate a suite of second-tier damaging agents, including the aldehydic lipid peroxidation products acrolein and 4-hydroxy-2-nonenal (HNE) (11, 12). Each of these reactive species can target specific sites in proteins, which, in turn, alter cell signaling pathways and cell fate decisions (11, 13, 14).

## Modification of proteins by oxidants and other reactive species

**Figure 1:**
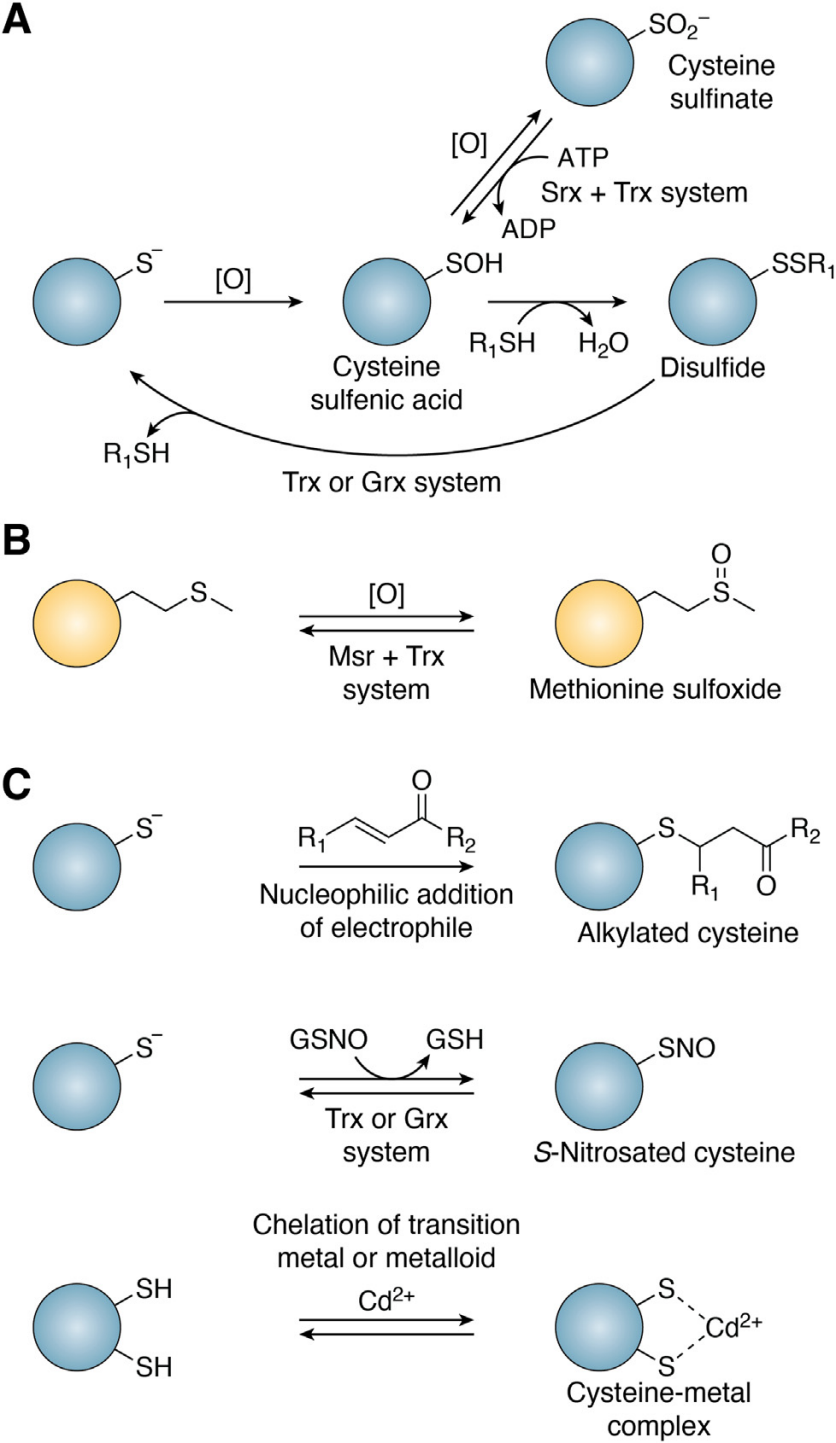
**Biologically relevant modifications to sulfur-containing amino acids.***A*, reversible oxidation of cysteine. Cysteine sulfenic acids form initially upon oxidation. These modifications can either undergo further oxidation to a sulfinate (*i.e.*, sulfinic acid) or condense with a thiol (R_1_SH) to form a disulfide. Sulfinates are reduced by sulfiredoxin (Srx), whereas disulfides can be reduced by either thioredoxin (Trx) or glutaredoxin (Grx). *B*, reversible oxidation of methionine. Upon oxidation of the methionine thioether, a sulfoxide is formed. This can be reduced by methionine sulfoxide reductases, which depend on the thioredoxin (Trx) system to be restored to their catalytically active form. *C*, other types of thiol modifications. Cysteines can undergo an electrophilic modification of the thiol, can react with nitrosating agents like *S*-nitrosoglutathione (GSNO), and can coordinate transition metals or metalloids.

The oxidants and other reactive species generated during cellular stress elicit many of their biological effects by damaging proteins, often by chemically modifying individual amino acid side chains (15, 16). These modifications can lead to altered protein activity under mild conditions but cause significant protein misfolding and aggregation during pronounced stress (17). While multiple proteinogenic amino acids are oxidizable, both sulfur-containing amino acids – cysteine and methionine – have specific reductases that reduce their commonly oxidized forms. Therefore, cysteine and methionine, distinct from other oxidizable amino acids, have been proposed to play a direct role in regulating protein activity and contribute to redox signaling *via* “redox switch” mechanisms (Fig. 1) (18, 19). Oxidizable cysteines can form disulfides within the same protein, between proteins, or with thiol-containing small molecules (*e.g.*, glutathione (GSH), coenzyme A, or cysteine) (Fig. 1*A*) (20, 21). These protein disulfides are far from inert, often undergoing thiol-disulfide exchange and direct reduction by the thioredoxin and glutaredoxin systems (20) (Fig. 1*A*). Yeast genetic studies indicate that the two disulfide reduction systems have some degree of functional redundancy (22). Both pathways draw their reducing power from NADPH and rely on partnering flavoenzymes, thioredoxin reductase, and glutathione reductase, respectively, to keep the corresponding “redoxin” protein in its reduced (*i.e.*, active) form (23, 24). In addition, the sulfinic acid form of cysteine is reparable by the ATP-dependent reductase sulfiredoxin (Fig. 1*A*) (25). While sulfiredoxin was thought originally to act solely on hyperoxidized forms of typical 2-Cys peroxiredoxins, recent evidence suggests that the enzyme may have a broader substrate specificity than originally anticipated (26). However, direct evidence of the reduction of sulfinic acids by sulfiredoxin in these alternative substrates has not yet been obtained. Methionine sulfoxides in oxidant-sensitive proteins, like proteins containing oxoforms of cysteine, can be reduced by particular reductases (Fig. 1*B*). In this case, a family of stereoselective methionine sulfoxide reductases (Msrs) employs either active site cysteine or selenocysteine residues to transfer electrons to their oxidized substrates, forming disulfide or mixed selenosulfide bonds in the process (27). Subsequently, the oxidized Msrs must be returned to their catalytically active form, with their reduction being catalyzed by thioredoxins (27).

The effects of reactive species on protein structure and function are not limited to oxidants. For instance, the thiol group of many oxidizable cysteine residues in proteins is susceptible to modification by other types of reactive species, due to its nucleophilicity and electron-rich character (Fig. 1*C*) (28). Soft electrophiles like those produced during lipid peroxidation (*e.g.*, HNE, acrolein) and xenobiotic metabolism can alkylate reactive cysteines that are surface-exposed (11, 29). Moreover, such cysteines can be modified by *S*-nitrosating agents and, with other electron-rich side chains, can be used to coordinate transition metals and metalloids (*e.g.*, cadmium, mercury, lead, arsenicals) at specific sites (Fig. 1*C*) (15, 30). As with oxidation, such modification can alter target protein activity and, in certain instances, results in structural perturbations within the target. While most types of thiol oxidation, metal binding, and *S*-nitrosation are reversible (18, 25, 31), modification of protein thiols by many organic electrophiles is considered terminal, requiring clearance of these damaged proteins and new production of the unmodified forms to rebalance homeostasis. Likewise, other amino acid side chains (*e.g.*, lysine, histidine, tyrosine) can be irreparably modified by oxidants, chlorinating agents, and electrophiles to alter protein structure and function (8, 15).

## Prominent protein targets of oxidants and other reactive species

Over the last two decades, several unbiased proteomic approaches have been employed to identify and characterize damage-prone proteins that are targeted by oxidants and other reactive species in multiple organisms, ranging from bacteria to multicellular eukaryotes (for examples, see (26, 32, 33, 34, 35, 36, 37, 38, 39, 40)). Such work has been enabled by the advent of bio-orthogonal chemistries that exploit both the unique reactivities of oxidized sulfur and incorporation of non-biological moieties (*e.g.*, alkynes and azides) for target enrichment, as well as advances in isotopic labeling procedures, mass spectrometry techniques, and data mining and bioinformatic analysis tools (41, 42, 43, 44). Since new tools and analytical methods have made possible the identification of previously uncharacterized types of modifications and new protein targets, the field of protein damage proteomics continues to advance and evolve. The targets of oxidants identified from proteomic screening approaches to date include key cytoskeletal proteins, metabolic enzymes, signaling enzymes, and numerous proteins in proteostasis networks (including several involved in protein biogenesis, folding, and clearance). Considerable overlap is observed between the oxidation-prone proteins and those possessing cysteines with heightened reactivity (often located in their active sites), as measured through quantitative proteomics methods (38). Thus, during oxidative stress and stress caused by other types of thiol-reactive species, multiple processes are affected simultaneously.

Of the protein targets of reactive species that have been identified, particular attention has been focused on the inactivation of metabolic enzymes in both glycolysis and the citric acid cycle. Of note, the glycolytic enzyme glyceraldehyde 3-phosphate dehydrogenase undergoes oxidation in the form of glutathionylation and persulfidation by hydrogen sulfide on its catalytic cysteine, leading to its inactivation (45, 46, 47, 48). Likewise, pyruvate dehydrogenase can be glutathionylated on its lipoic acid cofactor following the treatment of monocytic cells with the inflammatory stimulator lipopolysaccharide (49). In addition, several citric acid cycle enzymes and electron transport chain complexes are subject to oxidative inactivation. For instance, aconitase is inactivated upon reversible oxidation of cysteine residues that make up its FeS cluster (50), and the electron transport chain complex I protein NDUFS1 can be reversibly glutathionylated on cysteine 531 and cysteine 704 (51). These modifications collectively bring about a decrease in ATP synthesis during moderate to severe oxidative stress. Simultaneously, cells experience shifts in the overall proteostasis network, most notably oxidative inactivation of ATP-dependent folding mechanisms (*e.g.*, Hsp70, Hsp90) and oxidative activation of various chaperones with ATP-independent holdase activity, described more fully below.

## Chaperone systems in proteostasis

Molecular protein chaperones are evolutionarily conserved proteins and critical components of proteostasis networks that promote protein folding, refolding, or prevent protein aggregation under conditions when proteins are most vulnerable and susceptible to damage. Chaperones are classified based on their molecular mass or cellular function. Classified decades ago by protein size, there are at least five major chaperone groups including small heat shock proteins (sHsp, molecular weights ranging from 15 to 40 kDa), Hsp60s, Hsp70s, Hsp90s, and Hsp100s (52). In terms of molecular function, chaperones are colloquially categorized into two main functional types: foldases and holdases. Foldase chaperones assist in the folding of unfolded proteins or refold misfolded proteins *via* conformational cycling driven by ATP hydrolysis, whereas holdase chaperones bind to misfolded/unfolded proteins in a generally ATP-independent manner to prevent their aggregation or modulate proteolysis (53). The small heat shock proteins function exclusively as holdases (54), while other chaperones, such as Hsp70 and Hsp90, possess both foldase and holdase capacities (55). In addition, many chaperones require co-chaperone proteins (for example, J-proteins and nucleotide exchange factors) to modulate their activity by regulating ATP hydrolysis, as well as recognition, binding, and release of protein clients (56, 57). In general, these molecular chaperones and their co-factors play crucial roles in proteostasis by ensuring protein folding or refolding at the right place and right time, especially in stressful conditions.

## Effects of oxidative stress on chaperone function

### Oxidant-activated chaperones

Several proteins with non-chaperone functions are transformed into ATP-independent chaperones during oxidative stress, stabilizing misfolded proteins and preventing aggregation (Table 1). Upon exposure to strong oxidants (*e.g.*, HOCl, diamide), the zinc-binding protein Hsp33, which has zinc-coordinating cysteine residues that become oxidized to confer chaperone activity, has long served as the founding member of this class of chaperone. The mechanism of Hsp33 activation has been thoroughly reviewed elsewhere (17, 58, 59, 60). The oxidant-activated chaperone Hsp33 was first identified in bacteria. For many years, it was uncertain whether there was a eukaryotic equivalent of Hsp33. However, the yeast guanine nucleotide exchange factor Get3, which shares conserved CXC and CXXC motifs with Hsp33, likewise acquires ATP-independent chaperone activity when oxidized (61, 62). Transformation of Get3 into a chaperone is a multi-step event determined by the redox status of conserved cysteines 240 and 242 within the CXC motif. Oxidation of these cysteines triggers a conformational change, leading to Get3 oligomerization and concomitant acquisition of chaperone activity. Chaperone inactivation requires the reduction of the oxidized cysteines together with the binding of ATP to Get3, resulting in the release of protein clients to ATP-dependent foldases. Altering the chaperone activation/inactivation cycle by introducing serine substitutions at cysteines 240, 242 impairs resistance to oxidative stress, revealing the biological importance of the chaperone gain-of-function for Get3 (63). Other eukaryotic homologs of Hsp33 have recently been identified in algae (64) and in *Trypanosoma brucei* (65), underscoring the evolutionary importance of this oxidant-activated chaperone.

**Table 1:** Redox-regulated chaperones in eukaryotes

Chaperone class	Protein	Species	Normal role	Oxidative modification influencing chaperone activity	Impact of oxidation
Oxidant-activated chaperones					
	Get3	*S. cerevisiae*	insertion of tail-anchored proteins into ER membrane	disulfide in internal CXC motif	decreased ATP binding; acquisition of holdase activity
	TrypOx	*T. brucei*	unknown	not determined	acquisition of holdase activity
	algal Hsp33	*C. reinhardtii*	unknown	not determined	acquisition of holdase activity
	typical 2-Cys peroxiredoxins	many eukaryotes	peroxide detoxification; redox signaling	hyperoxidation of peroxidatic cysteine to sulfinate or sulfonate	acquisition of holdase activity
	α2-macroglobulin plasma albumin plasma fractions	*H. sapiens*	plasma proteins	N-chlorination	acquisition of holdase activity
Hsp70 family members					
	Hsp70 (cytosolic)	*S. cerevisiae; H. sapiens*	ATP-dependent protein folding; repression of Hsf1 activity	oxidation, electrophilic modification of various cysteines in NBD and SBD	acquisition of holdase activity
	Kar2/BiP	*S. cerevisiae*; *H. sapiens*	ATP-dependent protein folding in ER	oxidation of cysteine in nucleotide binding domain to a sulfenic acid and/or mixed disulfide with GSH	acquisition of holdase activity
Hsp70 nucleotide exchange factors					
	Fes1	*S. cerevisiae*	co-chaperone for Hsp70	oxidation of multiple methionines	impaired Hsp70 cycling
	Mge1/GrpEL1	*S. cerevisiae; H. sapiens*	co-chaperone for mitochondrial Hsp70	oxidation on at least one methionine	impaired mitochondrial Hsp70 cycling
Hsp90 family members					
	Hsp90α and β	*H. sapiens*	ATP-dependent protein folding and maturation	persulfidation, S-nitrosation electrophilic modification	acquisition of holdase activity

Peroxiredoxins (PRXs), thioredoxin-dependent peroxidase enzymes responsible for scavenging cellular peroxides, constitute a first line of defense for cells against rising levels of oxidants (66, 67, 68, 69). In addition to their antioxidant role, specific PRXs (particularly those of the typical 2-Cys class) act as multifunctional proteins, transducing redox signals to various oxidant-sensitive transcription factors and kinases (70, 71, 72, 73, 74). Moreover, during severe oxidative stress, some eukaryotic PRXs lose their peroxidase function and acquire holdase chaperone capacity (reviewed in (59, 75, 76, 77)). The peroxidatic cysteine in PRXs becomes hyperoxidized to a sulfinic acid, leading to PRX oligomerization into a high-molecular-weight form that possesses molecular chaperone activity (76). This modification was initially thought to be irreversible; however, sulfiredoxins were subsequently identified as a means of reducing the sulfinic acid formed upon PRX hyperoxidation, as noted earlier (78). The hyperoxidized form of the yeast PRX Tsa1 binds to client proteins and recruits Hsp70/Hsp104 chaperones to promote aggregate clearance (79), revealing the interplay between the PRXs and other chaperone systems.

While peroxiredoxins are thought to be principally hyperoxidized by their peroxide substrates, other proteins acquire chaperone activity by reacting with stronger oxidants (*e.g.*, HOCl), similar to Hsp33 and Get3. For instance, *Escherichia coli* RidA is transformed into a molecular chaperone when treated with HOCl. The treatment does not modify cysteines within RidA but instead reversibly *N*-chlorinates its positively charged amino acid residues, thereby leading to increased hydrophobicity of RidA and enhancing binding to unfolded proteins (80). Likewise, human α_2_-macroglobulin, a secreted protein that is transformed into a holdase chaperone in the presence of hypochlorite, transitions from the native tetramer to a dimer with enhanced surface hydrophobicity for unfolded protein binding (81, 82). Moreover, other human plasma components besides α_2_-macroglobulin (*e.g.*, albumin) exhibit chaperone-like activity when treated with HOCl (83). Collectively, the transformation of non-chaperone proteins into chaperones during oxidative stress is a common mechanism cells use to combat proteotoxicity, and it is likely that additional oxidant-activated (*i.e.*, gain of function) chaperones remain unidentified. Techniques for analyzing protein folding (84) and aggregation (85) may be employed to identify and validate these potential redox-activated chaperones, especially within a cellular context.

### Conversion of Hsp70 chaperones from foldases to holdases

**Figure 2:**
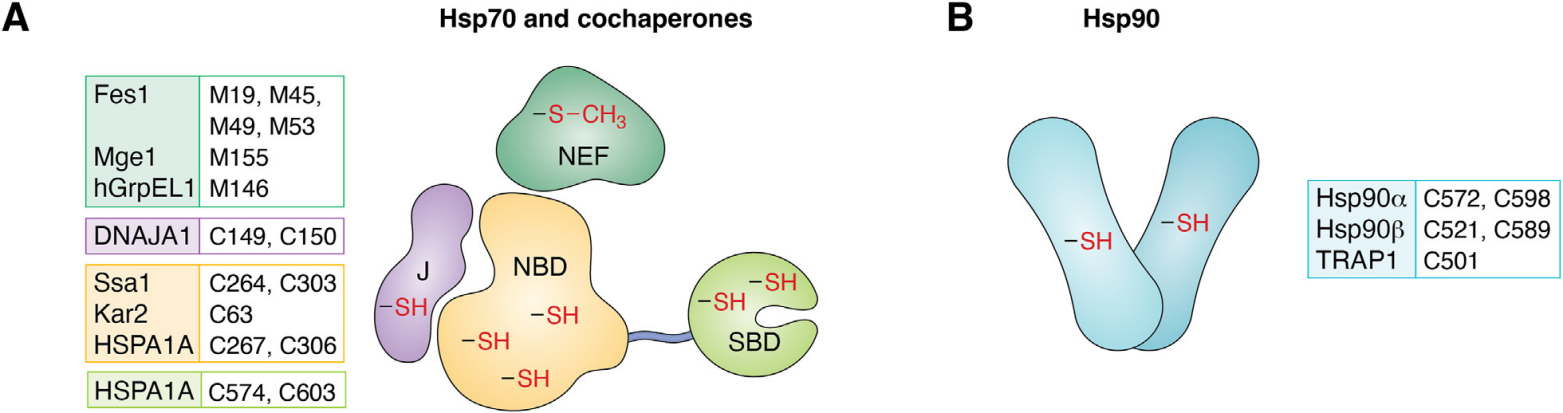
**The redox chaperone code.***A*, Hsp70 and cochaperone oxidation. Oxidation of cysteine (-SH) residues within the nucleotide-binding domain (NBD) or substrate-binding domain (SBD) of Hsp70s (Ssa1, Kar2, HSPA1A) transforms the chaperones from ATP-dependent foldases to ATP-independent holdases under oxidative stress. This redox switch is further facilitated by the oxidation of methionine (-S-CH_3_) residues on the Hsp70 cochaperone nucleotide exchange factors (NEFs - Fes1, Mge1, hGrpEL1). Additionally, the oxidation of cysteine residues on the J-domain protein cochaperone DNAJA1 titrates Hsp70 away from Hsf1, thereby inducing heat shock response activation. *B*, Hsp90 oxidation. Hsp90s (Hsp90α, Hsp90β, and TRAP1) are oxidized on cysteine (-SH) residues, leading to a decrease in ATPase activity and transition to ATP-independent holdases.

Members of the heat shock protein 70 (Hsp70) family play crucial roles in responses to many types of cellular stresses by participating in protein folding and refolding. They are highly modified at the post-translational level by multiple enzymes, with each modification dictating unique impacts on Hsp70 biological function, recently dubbed the “chaperone code” (86). In this section, we focus on the oxidative component of the Hsp70 chaperone code (Fig. 2*A*). In general, oxidative stress results in inhibition of ATP-dependent Hsp70 foldase activity. For example, we have identified that the yeast Hsp70 Ssa1 is targeted by thiol-reactive compounds (*e.g.*, cadmium sulfate, diamide, diethyl maleate, 15-deoxy-delta-12,14-prostaglandin J2), with cysteines 264 and 303 in the nucleotide binding domain serving as sites of modification (87). This modification leads to a decrease in ATP binding, hydrolysis, and protein foldase functions (88). Furthermore, oxidized Ssa1 fails to interact with heat shock transcription factor 1 (Hsf1), a key step in the regulation of the heat shock response in yeast (87, 88). These findings indicate that Ssa1 is a direct sensor of diverse thiol modifiers and suggest it could play a key role in redox signaling.

The cytosolic stress-induced human Hsp70 (HSPA1A) is also susceptible to oxidation, undergoing conversion from a foldase to a holdase. The redox cycler methylene blue oxidizes HSPA1A on cysteines 267 and 306 (orthologous to the previously mentioned reactive cysteines in Ssa1) to decrease ATP binding ability (89). Furthermore, overexpression of HSPA1A C267D and HSPA1A C306D mutants that mimic hyperoxidized cysteines decreases tau protein aggregation in HeLaC3 cells, implying a shift toward holdase activity (89). However, modification of some cysteines in Hsp70 family members does not decrease ATPase activity or enhance peptide binding upon oxidation. For instance, HSPA1A undergoes reversible glutathionylation on cysteines 574 and 603 in HeLa cells treated with diamide or glutathione (90). These cysteines are located in the carboxyl-terminal α-helical lid of the substrate binding domain that is unfolded upon cysteine glutathionylation. The unfolded α-helical lid structure binds to and blocks the substrate binding site. That binding also enhances the intrinsic ATPase activity of HSPA1A and disrupts binding to Hsf1 (90, 91). Since HSPA1A can be oxidized on five cysteine residues (90), different oxidation combinations may lead to different functional outcomes. Additionally, another human cytosolic Hsp70, HSPA8, also known as heat shock cognate 71 kDa protein (Hsc70), shares four cysteine residues with HSPA1A. However, due to its lower structural dynamics, HSPA8 exhibits significantly lower thiol reactivity than HSPA1A, which may result in differences in function and regulation (92). These findings highlight the diversity in the oxidative “chaperone code” for Hsp70 in protein folding.

Like its cytosolic counterpart, the ER-resident Hsp70 BiP (known as Kar2 in *Saccharomyces cerevisiae*) also switches from a foldase to a holdase upon oxidative imbalance in the ER. Cysteine 63 in Kar2 can be oxidized to form sulfenic acid (93) and undergoes reversible glutathionylation (94) (Fig. 2*A*). Various bulky and oxidomimetic mutations at this site lead to decreased ATPase activity and enhanced substrate binding (93, 94). In yeast, the Kar2 nucleotide exchange factor Sil1 facilitates glutathionylated Kar2 reduction, in addition to regulating the Kar2 folding cycle (95). Oxidative regulation of BiP activity appears to be evolutionarily conserved since cysteines within human BiP are also oxidized to increase polypeptide binding ability (96, 97).

Several Hsp70 cochaperones are also redox-regulated to influence the ATP-dependent foldase function of Hsp70, providing further support that Hsp70 folding is shut down during the initial phases of oxidative stress (98, 99, 100, 101, 102). For instance, the yeast cytoplasmic Hsp70 nucleotide exchange factor Fes1 is subject to oxidative stress-induced regulation on critical methionine residues. Fes1 can be oxidized on methionines 19, 45, 49, and 53, and these modifications are reversible by methionine sulfoxide reductases Mxr1 and Mxr2 (102). Fes1 methionine oxidation decreases nucleotide exchange activity, Hsp70 client release, control of peptide association, and interaction between Fes1 and Hsp70 (102). Likewise, the yeast mitochondrial nucleotide exchange factor Mge1, which interacts with Hsp70 to exchange ATP for ADP to facilitate the folding of client proteins, is oxidized on methionine 155 to a sulfoxide in the presence of H_2_O_2_ (98, 99). This oxidation induces structural changes in Mge1 (101), leading to a decrease in interaction with Hsp70 and a decline in Hsp70 ATPase and foldase activities, and is reversed by methionine sulfoxide reductase Mxr2. An oxidant-resistant mutation M155L in Mge1 prevents protein aggregation, suggesting that Mge1 oxidation regulates Hsp70 chaperone activity (99). Similarly, hGrpEL1, the human ortholog of Mge1, is oxidized on methionine 146 and is reduced by MsrB, the human ortholog of Mxr2, to recover Hsp70 ATPase activity. The hGrpEL1 M146L oxidation-insensitive mutation rescues the slow growth phenotype of the yeast *MXR2* knock-out mutant under oxidative stress in a humanized yeast model, indicating that regulation of mitochondrial Hsp70 nucleotide exchange factor through methionine oxidation is evolutionary conserved (100). Although a wealth of data demonstrate that the Hsp70 system appears to be heavily regulated at the redox level, several questions remain to be answered. What role, if any, do the cellular redox buffering pathways play in modulating the oxidation status of the chaperone and cofactors? Do thiol-reactive metabolites (*i.e.*, those with electrophilic centers) target Hsp70 to link metabolism and proteostasis? Finally, can we ontologically distinguish between oxidant “regulatory signaling” *versus* oxidant damage as it pertains to chaperones and other targets?

### Modification of Hsp90 by oxidants, electrophiles, and nitrosating agents

The heat shock protein 90 (Hsp90) family constitutes another group of highly conserved ATP-dependent molecular chaperones (103) that are subject to regulation by thiol-reactive molecules (Fig. 2*B*). Human Hsp90 undergoes oxidative modification (*e.g.*, persulfidation), electrophilic modification, and *S*-nitrosation (S-NO) (104, 105, 106, 107, 108, 109). For example, the human Hsp90α isoform is modified by the reactive lipid-derived electrophile 4-hydroxy-2-nonenal (HNE) on cysteine 572, causing a decrease in chaperone activity (110). Of the types of thiol-based modifications on Hsp90, perhaps *S*-nitrosation has been the most extensively studied.

Hsp90 *S*-nitrosation, while first discovered *in vitro* using recombinant human Hsp90α treated with NaNO_2_ or *S*-nitroso glutathione (GSNO), was further detected *in vivo* in endothelial cells treated with *S*-nitrosocysteine (CSNO) (104). This post-translational modification on cysteine 598 of Hsp90α leads to a decrease in chaperone ATPase activity (104). Human Hsp90β also undergoes *S*-nitrosation at cysteine 589, which corresponds to cysteine 597 in Hsp90α (106). Two adjacent cysteines are nitrosated differently in each isoform, suggesting a complex role for nitrosation in regulating Hsp90 function. Nitrosation on cysteine 589 in Hsp90β promotes the progression of fibrosis (106), while this same *S*-nitrosation event also promotes cardiac hypertrophy in a mouse model (107). Hsp90β is additionally *S*-nitrosated on cysteine 521, located in the middle domain of the protein, in the atherosclerotic aorta, and in oxidized LDL-treated endothelial cells (108). The middle domain of Hsp90 interacts directly with the cochaperone activator of Hsp90 ATPase activity 1 (AHA1), and nitrosation on cysteine 521 disrupts AHA1 binding, thus reducing Hsp90 ATPase activity (108). Switching Hsp90 from an ATP-dependent foldase to ATP-independent holdase status by *S*-nitrosation likely worsens pathology in cardiac fibrosis, cardiac hypertrophy, and atherosclerosis (106, 107, 108). In addition to cytosolic Hsp90, the mitochondrial Hsp90 molecular chaperone TRAP1 is subject to *S*-nitrosation on cysteine 501. Cysteine 501 nitrosation causes TRAP1 conformational changes, leading to decreased ATPase and chaperone activities while enhancing its proteasome-mediated degradation (111). It is therefore clear that both major chaperone families (Hsp70, Hsp90) undergo modifications (Fig. 2) that transform them from ATP-dependent foldases into ATP-independent holdases precisely when cellular ATP levels are decreased.

To gain a more comprehensive understanding of chaperone oxidation and its role in oxidative stress, future research should focus on comprehensively mapping redox-sensitive cysteine/methionine residues in chaperone systems; investigating the diverse types of cysteine oxidative modifications and their biological consequences on chaperone functions; determining the temporal and spatial dynamics of chaperone oxidations, including when, where, and to what extent these modifications occur in cells; identifying the protein clients of oxidized chaperones; exploring the role of chaperone oxidation in the pathogenesis, especially of age-related and neurodegenerative diseases; and investigating how chaperone systems collaborate with other cellular adaptive responses to maintain protein homeostasis.

## Cellular adaptive responses to proteotoxicity caused by oxidative stress

In the presence of oxidants, cells employ additional mechanisms besides chaperone functional switching to maintain cellular proteostasis. These responses include activating oxidative stress response genes to counteract changes in redox status and reduce oxidatively damaged proteins, suppressing global protein translation to decrease the production of nascent polypeptides which are vulnerable to damage, and enhancing the degradation of damaged proteins *via* both the proteasome and autophagy. Thus, maintaining cellular proteostasis is crucial for cell survival during oxidative stress (17, 112, 113, 114).

### Transcriptional activation of cytoprotective pathways

**Figure 3:**
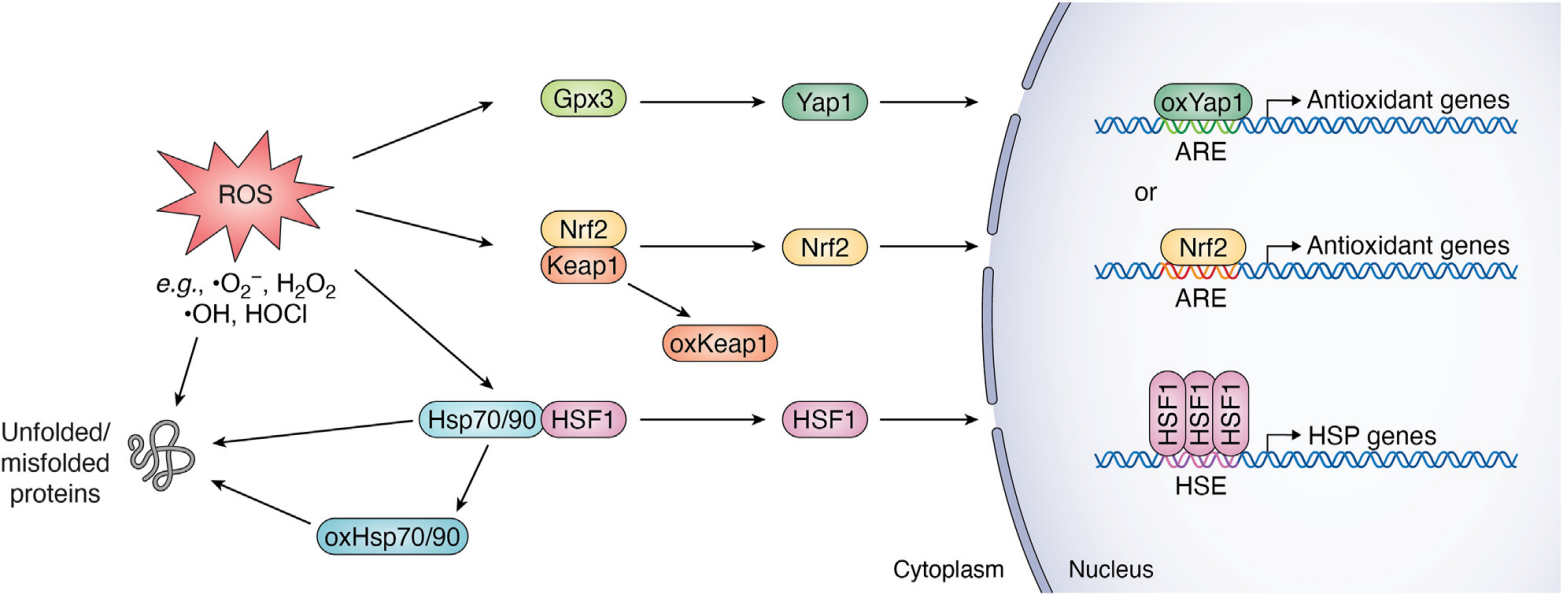
**Adaptive gene expression responses to oxidative stress that influence proteostasis.** In response to ROS, several parallel adaptive gene expression responses are activated. These include the antioxidant response (controlled by the transcription factor Yap1 in *Saccharomyces cerevisiae* and the transcription factor Nrf2 in mammals) and the heat shock response (controlled by the transcription factor Hsf1 in many different eukaryotic species). Proteins sensitive to oxidation that are thought to play a role in the regulation of these transcription factors are indicated.

During oxidative stress, the expression of genes encoding components of the glutathione and thioredoxin systems, peroxidases, cysteine transport proteins, and enzymes that regulate NADPH production, is increased (Fig. 3) (112). Rapid mobilization of these oxidant response factors helps to eliminate ROS and provide time for cellular remodeling of protein synthesis and refolding activities, reduction of reversibly oxidized proteins, and clearance of oxidatively-damaged proteins (reviewed in (113, 114, 115, 116, 117)). In *S. cerevisiae*, the transcription factor Yap1 regulates transcription of most antioxidant genes (115). In the presence of H_2_O_2_, Yap1 is oxidized on cysteines 303 and 598 by the hydroperoxide receptor glutathione peroxidase 3 (Gpx3), which prevents recognition by the nuclear exportin Crm1 and results in nuclear retention and increased expression of its target genes (118, 119). In mammalian cells, the Nrf2 transcription factor serves as the master regulator of antioxidant defense genes (116). Under normal growth conditions, Nrf2 activity is repressed by binding KEAP1, a prominent signaling target of both oxidants and electrophiles. During oxidative stress, several reactive thiols of cysteine residues within KEAP1 are oxidized, leading to KEAP1 conformational changes that prevent it from targeting Nrf2 for degradation. Free Nrf2 then localizes to the nucleus to activate the transcription of its target genes (116, 117, 120). KEAP1 acts as an oxidative stress sensor with its cysteines being oxidized by ROS and HOCl (116, 120), dietary electrophiles (organosulfur compounds, α,β-unsaturated moiety-bearing compounds) (121, 122), endogenous reactive metabolites (*e.g.*, oxidized *N*-acetylserotonin, methylglyoxal) (123, 124), and glycolysis and citric acid cycle intermediates (125, 126), to activate Nrf2. Intriguingly, a link exists between chaperone systems and the Nrf2-KEAP1 complex. Hsp90-KEAP1 interaction was detected *in vivo* (127), and Nrf2 physically and genetically interacts with Hsp90 in a humanized yeast model (128). Nrf2 activation by oxidative stress is likely promoted by Hsp90 to ensure that Nrf2 is maintained at a high enough level and properly localized to activate gene transcription, given the extensive disordered regions in the protein (128).

The heat shock response (HSR), another key adaptive transcriptional response regulated by the evolutionarily conserved heat shock transcription factor Hsf1, is activated by elevated temperatures and many other proteotoxic stresses, including oxidative stress (Fig. 3) (112, 129, 130, 131, 132, 133). Hsf1 regulates the transcription of genes encoding molecular chaperones (*i.e.*, heat shock proteins) and other cytoprotective genes (112, 134). The increase in molecular chaperone expression minimizes stress-induced protein aggregation, supports protein refolding, and stimulates the degradation of terminally damaged proteins (112, 135, 136). Under normal growth conditions, heat shock proteins Hsp70 and Hsp90 bind to Hsf1 to form a stable complex that inhibits Hsf1 oligomerization and activity (136, 137, 138, 139, 140, 141). The binding of Hsp90 to Hsf1 was observed in mammalian cells and is thought to be a transient interaction, while it is not detected in yeast (137, 139, 140). Conversely, Hsp70 binds to Hsf1 more stably, and multiple lines of evidence identify it as the major repressor of Hsf1 (136, 138, 140, 141, 142, 143).

Multiple models have been proposed for how the derepression of Hsf1 occurs during the stress caused by reactive molecules like oxidants and electrophiles. In one such model, misfolded or unfolded proteins that accumulate immediately after protein damage binds to and titrate Hsp70 and Hsp90 away from Hsf1. This shift of chaperone binding away from Hsf1 to client proteins could derepress Hsf1, allowing it to upregulate its target genes (136, 138, 141, 143, 144). Such a mechanism of titration of Hsp70 by misfolded/unfolded proteins was first observed with Hsf1 in yeast (138, 141) and has since been confirmed in mammalian cells (143). Hsp70 titration is also triggered by its binding partner Hsp40. For instance, in the mitochondrial unfolded protein pathway, mitochondrial ROS oxidize the human Hsp40 DNAJA1 on cysteines 149 and 150, which recruits Hsp70 away from Hsf1 to activate HSR (145). Importantly, Hsp70 titration by misfolded/unfolded proteins requires the concurrence of active protein translation, as nascent chain polypeptides are the most vulnerable to misfolding and aggregation during stress (146). Notably, orphan ribosomal proteins, which account for nearly 50% of translating proteins in dividing cells, are susceptible to misfolding during oxidative stress and trigger the HSR (144, 147). Activation of Hsf1 involves several key steps: nuclear localization, trimerization, hyperphosphorylation, and binding to the heat shock element (HSE) in the promoters of target genes to drive transcription (130). Generally, HSR activation regulation converges on releasing Hsf1 by disrupting its interaction with chaperones.

In addition to Hsp70 titration by its substrates, Hsp70 release of Hsf1 can be influenced by post-translational modifications to either Hsp70 or Hsf1 itself (129, 130, 132). We demonstrated that thiol-reactive molecules modify cysteine 303 in the yeast Hsp70 Ssa1 *in vivo* (87) and a mutant version of Ssa1 bearing two substitutions that mimic cysteine hyperoxidation (C264D, C303D) destabilized its interaction with Hsf1, leading to constitutive activation of the heat shock response (88). Separately, a decrease in monomethylation of human Hsp70 at arginine 469 by Jumonji domain-containing protein 6 (JMJD6) is thought to disrupt the binding between Hsp70 and Hsf1. Interestingly, JMJD6 transcription is activated by Hsf1 in a positive feedback loop, despite the absence of HSEs in the JMJD6 promoter (148). Since Hsp70 binds to Hsf1 through its substrate-binding domain (140, 141, 143), any other redox-induced modifications in this domain (*e.g.*, for example, the cysteine glutathionylation of human Hsp70 HSPA1A) could also potentially activate Hsf1. Regarding Hsf1 modifications, H_2_O_2_ can induce disulfide bond formation in human Hsf1, which leads to Hsf1 trimerization (134). In addition, Hsf1 hyperphosphorylation is associated with its DNA binding and transcriptional activity, and mimicking constitutive phosphorylation results in prolonged HSR activation (129, 130). It is believed that Hsf1 phosphorylation is crucial for fine-tuning its activity. For instance, during mitochondrial stress, Hsf1 dephosphorylation has recently been reported, and that hypophosphorylated Hsf1 selectively activates the transcription of only certain small heat shock proteins (*i.e.*, not the entire Hsf1 regulon) (149). Several other post-translational modifications of Hsf1 (*e.g.*, acetylation, SUMOylation, and ubiquitination) have been documented (129, 130). However, the biological function of these Hsf1 modifications is not yet fully understood, and further research is needed to determine the effect these modifications have on the heat shock response during oxidative stress.

### Redox regulation of translation and ribosomes

**Figure 4:**
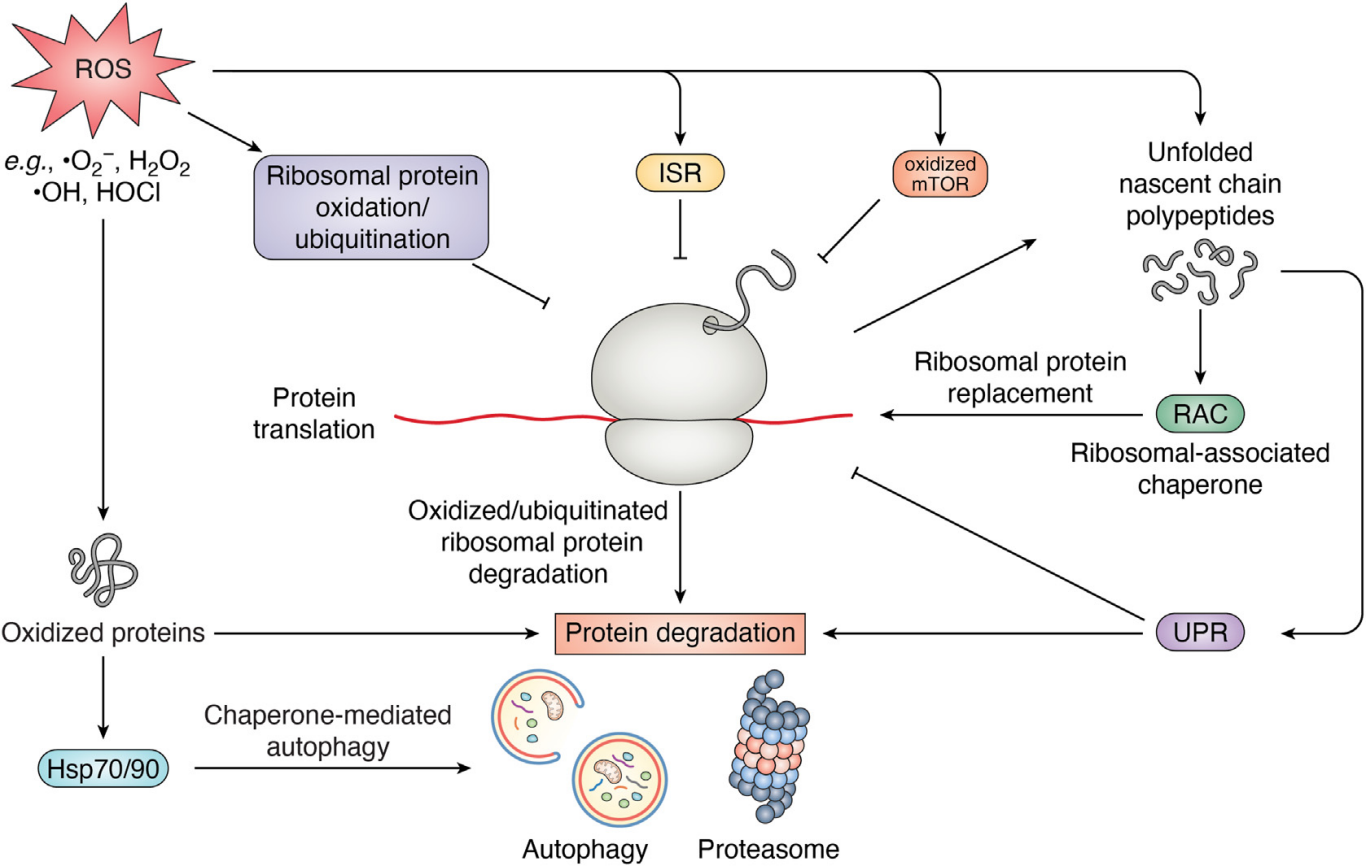
**Impact of oxidative stress on protein biogenesis and damaged protein clearance.** ROS regulate both protein translation and protein clearance through several distinct mechanisms. For translation, ROS directly modify ribosomal protein components or trigger signaling pathways that inhibit translation. Likewise, the turnover of proteins damaged during oxidative stress occurs through both proteasomal degradation and different types of autophagy.

Global repression of translation during oxidative stress comprises another important event utilized by cells to minimize oxidative damage to nascent chains and conserve energy as cellular ATP levels drop due to the oxidative inhibition of metabolic enzymes (112, 150, 151, 152, 153, 154, 155). During oxidative stress, multiple translation components, including mRNAs, RNA binding proteins, and translation initiation factors, localize to specific foci (*e.g.*, P-bodies, stress granules), priming these factors for rapid reactivation when redox status is attenuated (156, 157). The formation of these structures is likely due to the oxidation of several core ribosomal components (154). Additionally, the integrated stress response (ISR) pathway is activated to shut down translation initiation (Fig. 4). This pathway detects stress through the kinases PERK, GCN2, PKR, and HRI, which phosphorylate a single serine residue on eukaryotic translation initiation factor eIF2. This phosphorylation blocks the function of guanine nucleotide exchange factor eIF2B, leading to a decrease in protein translation (158). PERK, in particular, responds to unfolded proteins in the ER during oxidative stress (159), while the mechanisms by which the other three kinases receive redox signals remain to be elucidated. Recent reviews focusing on the ISR and oxidative stress can be found elsewhere (132, 158, 160, 161). In addition to the ISR, translation is influenced by the mechanistic target of rapamycin (mTOR) kinase that inactivates elongation initiation factor 4E-binding protein 1 (4E-BP1) to promote translation initiation (160, 162). However, oxidative stress induces the formation of intermolecular disulfide bonds at cysteine 1483 in mTOR, resulting in its inhibition and subsequent repression of translation (163). Further details on how mTOR inhibition under oxidative stress halts translation can be found in recent reviews (132, 164).

While the translation machinery is repressed, newly synthesized or nascent polypeptides fail to fold into their native states under oxidative stress conditions. A subset of these polypeptides accumulates in the ER lumen or mitochondria, which triggers activation of the ER unfolded protein response (UPR) or the mitochondrial unfolded protein response (UPR^mt^) pathways, respectively. These responses aim to restore cellular homeostasis or, in cases of prolonged stress, trigger apoptosis (165, 166). Multiple components of the ER and mitochondrial UPR pathways undergo redox modifications at cysteine residues during oxidative insults (167) as well as the interplay between the UPRs and redox signaling is thoroughly summarized in the recent reports (132, 166, 167, 168).

Following oxidative injury, ribosomes must be repaired to restore translation. Nascent ribosomal proteins aggregate upon oxidation, necessitating assistance from a distinct set of ribosome-associated chaperones (Fig. 4) (154, 169, 170, 171). For example, upon H_2_O_2_ treatment, Rps26 is oxidized on cysteine 74 and cysteine 77, while Rpl10 is oxidized on cysteine 105 (172). These oxidized proteins are terminally damaged and must be replaced with newly synthesized ribosomal proteins by the chaperones Tsr2 and Sqt1, respectively, to maintain ribosome integrity (172). In addition, another 11 ribosomal proteins have their own specific chaperones (171) and among them the proteins Rps2, Rps3, Rpl3, Rpl4, and Rpl23 are oxidized under oxidative stress (172), suggesting that this repair mechanism might be applied to these proteins as well. Cysteine 105 in Rpl10 and the chaperones Tsr2 and Sqt1 are evolutionarily conserved from yeast to humans (172), underscoring both the vulnerability and the need for this chaperone-directed repair pathway.

Ribosomal proteins are subject to ubiquitination under oxidative stress. For example, several yeast ribosomal proteins are ubiquitinated *via* the K48-linked polyubiquitination (K48-Ub) mechanism following hydrogen peroxide treatment (173). K48-Ub attachment serves to target proteins for degradation by the proteasome (174, 175, 176). In contrast, treating yeast cells with H_2_O_2_ caused the accumulation of K63-linked polyubiquitination (K63-Ub) on ribosomal proteins that reside at the head of the 40S ribosome subunit (177, 178). This K63-ubiquitin modification is catalyzed by the E2 ubiquitin-conjugating enzyme Rad6 (179), while the deubiquitinating enzyme Ubp2 is deactivated under oxidative stress (177). The K63-Ub modification of ribosomal proteins transiently inhibits translation by impacting translation factor binding, polysome assembly, and translation elongation (178, 180, 181). Suppression of K63-Ub addition increases cell sensitivity to oxidative stress (177), suggesting that this modification plays a role in redox regulation of translation. In sum, oxidative stress can differentially trigger the degradation of ribosomal proteins *via* K48-Ub addition or regulate their functions *via* K63-Ub conjugation, providing flexibility in the translational response to oxidative insult.

The extent of translation repression during oxidative stress remains unclear, as translation of stress response genes is required to mount an effective response. Are there specific translation pause sites that emerge during oxidative stress to allow time for nascent polypeptides to fold or be transported? Future studies should also focus on the cooperation of redox-regulated ISR, UPR, and ribosomes in translation, as well as the identification of any related unknown oxidative stress sensors. Additionally, the effect of oxidative stress-induced K63-polyubiquitination on the 60S ribosome subunit should be explored. Furthermore, it remains to be determined whether ribosome-associated chaperones are also redox-regulated or merely play a role in shielding nascent polypeptide chains from oxidative insult.

### Oxidative stress-induced autophagy

In addition to activating oxidative stress-responsive genes and halting global protein translation, cells must eliminate oxidized proteins that are irreversibly damaged by targeting them for degradation either by the proteasome or by the autophagy/lysosomal machinery (Fig. 4). Proteasomes, large complexes that contain several distinct proteolytic activities, play a crucial role in the regulated degradation of cellular proteins. Degradation of oxidized proteins by the proteasome has been extensively reviewed (17, 182, 183). On the other hand, the process of autophagy removes nucleic acids, proteins, lipids, and cellular organelles through lysosome-mediated degradation, ensuring cellular homeostasis. The three main types of autophagy are microautophagy, macroautophagy, and chaperone-mediated autophagy (CMA) (184). The connections between oxidative stress and both microautophagy and macroautophagy have been highlighted in recent reviews (132, 185, 186, 187, 188, 189, 190). We specifically focus here on the intersection between oxidative stress and CMA (191, 192). CMA degrades proteins with a specific consensus motif (KFERQ) and some variations caused by post-translational modification. The motifs are recognized and bound by cytosolic Hsc70 (Hsp70) (191). Hsc70-protein substrate complexes interact with the lysosomal membrane receptor, the lysosome-associated membrane protein type 2A (LAMP2A), and trigger LAMP2A polymerization to form a complex that helps transport the target protein into the lysosome with the help of lysosomal Hsc70 (191, 192). Hsc70 is localized to the lysosome membrane, where it forms a complex with Hsp90, Hsp40, Hsp70-Hsp90 organizing protein HOP, and the Hsp70-interacting protein HIP. This complex facilitates the unfolding of the target substrate before lysosomal translocation (193). CMA activity is maintained at basal levels during homeostasis but is highly activated upon oxidative stress (194). Oxidative stress can increase the presence of Hsc70 and Hsp90 at the lysosome and up-regulate the expression of LAMP2A, which enhances CMA and uptake of misfolded proteins for degradation (194). CMA also degrades KEAP1 upon oxidative stress, allowing Nrf2 to up-regulate LAMP2A gene expression through a feed-forward loop (195). In addition, in aging cells, where there is increased oxidative burden, restoration of CMA in the liver improves cellular maintenance and overall hepatic function while loss of CMA increases proteotoxicity in an aging mouse model (196, 197). Overall, the chaperone system not only collaborates with the proteasome system but also works closely with autophagy to remove oxidatively damaged proteins, with clear physiological relevance. It is still uncertain whether chaperone oxidation enhances lysosomal localization or influences binding to the consensus motif of protein clients. Moreover, the CMA receptors that receive redox signals need to be identified, along with the mechanisms by which autophagy collaborates with the proteasome system to remove oxidatively damaged proteins.

## Conclusion

Organisms regularly encounter rising oxidant levels, originating from both internal and external sources that may cause redox dysregulation. This imbalance disrupts protein homeostasis, putting a broad range of cellular functions at risk. Under such oxidative stress, proteins are subjected to both permanent damage and reversible oxidation on the thiol groups in cysteines or the thioether groups within methionine residues. These thiol modifications, surprisingly, appear to play important regulatory roles, altering or activating protein functions. Notably, they occur at specific sites across a wide range of proteins involved in the proteostasis network, including in oxidative-stress response transcription factors, ribosomal proteins, protein degradation systems, and chaperone systems. Within the chaperone systems, thiol modifications target not only major chaperones like Hsp70 and Hsp90, converting them from ATP-dependent foldases into ATP-independent holdases, but also co-chaperones including nucleotide exchange factors (Table 1) (Fig. 2). Tellingly, oxidation of co-chaperones in many cases results in the same outcome as oxidation of the chaperones themselves—promoting energy-independent holdase activity in the corresponding partner chaperone. These effects are consistent with an evolutionarily conserved cellular strategy to safely pause protein metabolic activity during oxidative insult when ATP levels are insufficient to allow proteome-wide refolding by the highly abundant chaperone systems. Intriguingly, cells take yet another approach to preserve proteostasis—transforming non-chaperone proteins into molecular chaperones through thiol modification. Therefore, the diverse thiol modifications on proteostasis-regulating proteins highlight the intricate cellular response to oxidative proteotoxic stress. While our understanding of these modifications continues to advance, several key questions remain. What other proteostasis factors are subject to thiol-based regulation by oxidants and other reactive species? Further, how do cells orchestrate these responses to effectively mitigate oxidative stress, and how does this occur at the tissue and organ level in humans? To address these questions, recent technological advances in thiol biology have emerged (reviewed in (44)). These advances include curated cysteine modification databases such as iCysMod (198), CysModDB (199), and CysDB (200); the direct cysteine-glutathionylation quantification method G-ICAT (glutathione-based isotope-coded affinity tag) (201); the H_2_O_2_ sensor HyPer7 fused to proteins to study redox biology at the nanoscale (202); and the targetable reactive electrophiles and oxidants (REX) technology to precisely and spatially identify redox sensors (203). As cumulative oxidative damage is a clear driver of biological aging, it will be paramount to translate these findings to the organismal level and ultimately to patients to identify therapeutic interventions in relevant pathologies.

## Data availability

All supporting data are provided within the manuscript, supplementary data and supplementary tables.

## Conflict of interest

The authors declare the following financial interests/personal relationships which may be considered as potential competing interests.

Kevin A. Morano reports financial support was provided by the National Institute of General Medical Sciences. If there are other authors, they declare that they have no known competing financial interests or personal relationships that could have appeared to influence the work reported in this paper.
